# INSnet: a method for detecting insertions based on deep learning network

**DOI:** 10.1186/s12859-023-05216-0

**Published:** 2023-03-06

**Authors:** Runtian Gao, Junwei Luo, Hongyu Ding, Haixia Zhai

**Affiliations:** School of Software, Henan Polytechnic University, Jiaozuo, 454003 China

**Keywords:** Structural variation, Insertion, Deep learning, Depthwise separable convolutional network, Gated recurrent unit

## Abstract

**Background:**

Many studies have shown that structural variations (SVs) strongly impact human disease. As a common type of SV, insertions are usually associated with genetic diseases. Therefore, accurately detecting insertions is of great significance. Although many methods for detecting insertions have been proposed, these methods often generate some errors and miss some variants. Hence, accurately detecting insertions remains a challenging task.

**Results:**

In this paper, we propose a method named INSnet to detect insertions using a deep learning network. First, INSnet divides the reference genome into continuous sub-regions and takes five features for each locus through alignments between long reads and the reference genome. Next, INSnet uses a depthwise separable convolutional network. The convolution operation extracts informative features through spatial information and channel information. INSnet uses two attention mechanisms, the convolutional block attention module (CBAM) and efficient channel attention (ECA) to extract key alignment features in each sub-region. In order to capture the relationship between adjacent subregions, INSnet uses a gated recurrent unit (GRU) network to further extract more important SV signatures. After predicting whether a sub-region contains an insertion through the previous steps, INSnet determines the precise site and length of the insertion. The source code is available from GitHub at https://github.com/eioyuou/INSnet.

**Conclusion:**

Experimental results show that INSnet can achieve better performance than other methods in terms of F1 score on real datasets.

**Supplementary Information:**

The online version contains supplementary material available at 10.1186/s12859-023-05216-0.

## Background

Differences between individuals are usually manifested as single nucleotide variations (SNVs), small insertions and deletions (indels; < 50 bp), and structural variations (SVs; ≥ 50 bp) [1]. SVs are insertions, deletions, duplications, inversions, translocations, and combinations of these categories that are longer than 50 bp. Although single nucleotide polymorphisms (SNPs) are the most common genomic variation, SVs have a greater impact than any other class of variation [2]. Many studies have shown that SVs have a considerable impact on human diseases and disorders, such as cancer [3] and schizophrenia [4]. Insertions are an important category of SVs that can cause serious diseases. For example, X-linked dystonic Parkinson’s disease (XDP) is a neurodegenerative disease caused by retrotransposon insertion [5]. Independent mutation of L1 insertion into exon 14 of the factor VIII gene causes haemophilia [6]. Alu element insertion ultimately results in myotonic dystrophy type 2 (DM2) [7]. Insertions are critical to our understanding of human genetics and precision medicine. It can be combined with clinical features to help classify diseases and predict the correlation between drugs and diseases [8, 9].

Sequencing technology is very important for the detection of SVs. This technology has included Sanger sequencing [10], next-generation sequencing (NGS) technology [11], long-read sequencing [12], and circular consensus sequencing (CCS) [13]. NGS technology has a high accuracy rate, which can reach 99%, and the sequencing cost is low. However, the sequencing length is short, only 150–500 bp. As a result, it is still difficult to obtain high-quality assembly or alignment results for highly repetitive gene regions when using NGS technology. At the same time, second-generation read technology requires additional processing of the sequencing samples, and these operations will introduce bias into the sequencing results. With the rapid development of long-read sequencing technology, the third-generation sequencing technologies of Pacific and Oxford Nanopore Company provide opportunities for more comprehensive detection of SVs. The average length of sequences resulting from third-generation sequencing is more than 10 kbp, but this approach has an error rate as high as 5–20%. The latest technology, CCS, has improved the accuracy of single-molecule real-time (SMRT) sequencing (PacBio). CCS technology produces long reads with an average length of 13.5 kilobases (kb) and an accuracy greater than 99% [13]. With advances in sequencing technology, sequencing data has enabled the computation of predicting protein–protein interactions (PPIs) [14] and gene co-expression module detection [15] have also developed significantly, which is of great significance in biology.

Many methods have been developed to call SVs based on different sequencing technologies. These methods can be commonly divided into the following two categories.

(i) Methods based on short reads produced by NGS technology. There are many SV callers that use short reads, such as DELLY [16], LUMPY [17], BreakSeek [18], SIns [19] Manta [20], CNVnator [21], PEMer [22] and BreakDancer [23]. These methods usually call SVs by read depth, discordant read pairs and split reads from alignments between short reads and the reference genome. Read depth refers to the average number of reads mapped to one position in the reference genome. If the alignment distance and orientation of one read pair are different from the expected values, the read pair is considered discordant. A split read is a read aligned with several parts. Many SV caller, such as CNVnator, PEMer, BreakDancer only use one of the above features. This greatly limits the detection of SVs. And DELLY, LUMPY, BreakSeek, SIns, Manta all use two or three of the above features to effectively detect SVs. DELLY, LUMPY, and BreakSeek all use combined discordant read pairs and split read methods to effectively detect SVs.
(ii) Methods based on long reads produced by third-generation sequencing technologies. Long reads can span long regions in the reference genome, which can facilitate complex variant detection. Many methods take advantage of long reads to call SVs. To overcome the high error rate of the obtained sequences, Sniffles [24] adopts a new SV scoring scheme to call SVs based on the size, location, type, and coverage of candidates. When calling insertions, Sniffles uses the CIGAR string and MD to find the relevant region. SVIM [25] detects SVs by the graph clustering method. It mainly finds insertions through inter-alignment and intra-alignment. Inter-alignment involves finding the insertion through the CIGAR string. Intra-alignment reveals variant points through split read information. CuteSV [26] uses multiple extraction methods to comprehensively collect signatures of various SVs; specifically, it designs clustering and refinement methods to accurately distinguish SV features from heterozygous SVs. It also mainly extracts CIGAR strings and split read information to call insertions. These tools can use long-read alignment files generated by aligners such as NGMLR, minimap2, pbmm2, and BWA-MEM as input to call SVs. PbSV is a SV caller for PacBio single molecule real-time sequencing (SMRT) reads. It uses split reads and intra-signatures to detect SVs. NanoSV [27] is a SV caller for nanopore data. It mainly uses split reads to detect SVs.

Although current traditional methods have greatly advanced the detection of SVs, they still have some problems. For SV callers based on short reads, although the short reads have high accuracy, the length is relatively short. It is difficult to span the insertion region with a large length. Therefore, there are some problems about large insertion detection. And the results show that DELLY and BreakSeek have better performance in small SVs, and LUMPY. For SV callers based on long reads, although the average length of long reads is more than 10 kbp, but long reads have a high error rate. How to detect SVs in long reads with high error rate is very important. And it is significant to distinguish sequencing errors from SV sites. And, none of these advanced tools fully solve the problem of large insertions [28].

Deep learning can extract more significant features to solve complex problems and has been used to detect SVs. DeepVariant [29] uses a convolutional neural network (CNN) to call SNPs and small indels and outperforms all state-of-the-art variant callers. DeepSV [30] uses a new visual sequence read method to call long deletions through deep learning. However, both callers use short-read data to detect variants.

As deep learning networks can learn very large and more complex features from large datasets more efficiently than ever before, they have achieved great success in many fields. The strong fitting ability of deep learning networks is expected to improve the detection of insertion regions. In this work, we introduce INSnet, a method for detecting insertions based on a deep learning network. INSnet uses alignments between long reads and the reference genome as input and uses depthwise separable convolution [31], an attention mechanism, and a bidirectional gated recurrent unit (GRU) network to effectively detect insertion regions of different sizes [32]. Experimental results show that INSnet achieves better insertion detection results than Sniffles, SVIM, and cuteSV. Moreover, INSnet achieves a high F1 score on different real datasets.

## Methods

**Fig. 1:**
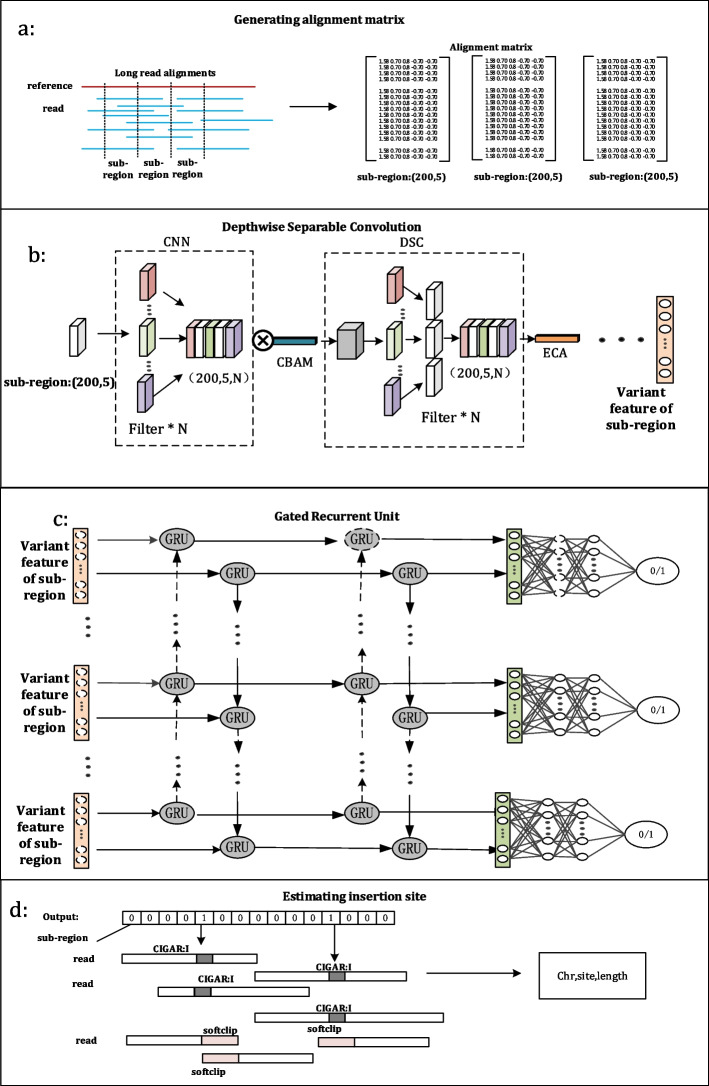
The workflow of INSnet. **a** Generating the alignment feature matrix. **b** Extracting variant features. **c** Determining sub-regions containing insertions. **d** Estimating the insertion site and length

INSnet is an insertion detection method based on long reads and a deep learning network that can effectively detect insertions. It is mainly divided into four steps. (i) Generating the alignment feature matrix. INSnet uses the alignment file between long reads and the reference genome as input. It splits the reference genome into sub-regions of the same length. For each sub-region, it generates an alignment feature matrix. (ii) Extracting variant features. INSnet adopts a depthwise separable convolutional network and two attention mechanisms to obtain the variant feature for each sub-region. (iii) Determining sub-regions containing insertions. INSnet uses the variant features among continuous sub-regions through the bidirectional GRU neural network to determine the sub-regions that contain insertions. (iv) Estimating the insertion site and length. For each sub-region detected in the previous step, INSnet finds the exact insertion site and length according to the alignments on the sub-regions. The four steps are shown in Fig. 1.

### Generating the alignment feature matrix

INSnet uses the alignment file as input to extract alignment features. INSnet first divides the reference genome into *n* sub-regions with the same length of 200 bp. For each site in a sub-region, INSnet extracts five features and generates a 5-tuple (read_depth_, read_ls_, read_rs_, read_sr_, read_ic_). Read_depth_ denotes the read coverage of the site. Read_ls_ denotes the count of left soft-clip breakpoints at this site (An example is shown in the Additional file 1: Fig. S1). Read_rs_ denotes the count of right soft-clip breakpoints at this site (An example is shown in the Additional file 1: Fig. S2). Read_sr_ denotes the number of split alignments at this site. Read_ic_ denotes the count of the insertion in the CIGAR string (‘I’ operation in the CIGAR string).

For a sub-region, INSnet can generate an alignment feature matrix with 200 rows and 5 columns. If the length of a sub-region is less than 200 bp, the corresponding column of the matrix is filled with 0. Then for each row of an alignment feature matrix, INSnet pre-processes it using Z score normalization [33]. The z score makes the values in the same column have a mean of 0 and a standard deviation of 1. This operation preserves the shape properties of the original features, improves the training speed of the deep learning model, and increases the accuracy. The Z score can be represented by the equation:

1 $$z - {\text{score}}\left( {\text{x}} \right) = \frac{{{\text{x}} - {\text{Mean}}}}{{{\text{Standard}} - {\text{Deviation}} }}$$

### Extracting variant features

Due to the large amount of data, directly using the traditional fully connected neural network will require a substantial number of parameters and increase the computational cost. INSnet uses a CNN to extract variant features for each alignment feature matrix. INSnet first uses only one layer of traditional convolution [34] and max pooling to extract variant features. Then, INSnet uses a depthwise separable CNN [31]. Compared with traditional convolution, the depthwise separable convolution consists of two steps: depthwise convolution and pointwise convolution. Depthwise convolution uses a convolution kernel for each channel of the input feature map and then splices the outputs of all convolution kernels to obtain its final output. Pointwise convolution is a 1 × 1 convolution that is used to change the number of feature channels. Depthwise separable convolution can further reduce the number of parameters.

The convolution operation extracts informative features through spatial information and channel information. Therefore, INSnet uses two attention mechanisms, the convolutional block attention module (CBAM) [35] and efficient channel attention (ECA) [36], to further process the features. The CBAM uses channel and spatial attention modules to increase the expressiveness of features through an attention mechanism. The specific calculation formula is shown in (2).

2 $$\begin{array}{*{20}l}{{\text{F}^{\prime}}={\text{C}}\left({\text{F}}\right)\otimes{\text{F}}}\hfill\\{{\text{F}^{\prime\prime}}={\text{S}}\left({{\text{F}^{\prime}}}\right)\otimes{\text{F}^{\prime}}}\hfill\\\end{array}$$

*F* refers to the feature after convolution and max pooling, and F″ refers to the result of passage through the 1D channel attention module C and the 2D spatial attention module S. $\otimes$ indicates elementwise multiplication.

The CBAM uses average pooling and max pooling to aggregate features and uses two-dimensional convolution to calculate spatial attention, which is computationally expensive. Therefore, after using a CBAM module, INSnet uses the ECA module, which avoids dimension reduction and captures cross-channel information in an efficient way. ECA is efficiently implemented using only one fast 1D convolution of size *k*. The specific calculation formula is as follows:

3 $${\text{w}} = \sigma \left( {{\text{C1D}}_{{\text{k}}} \left( {\text{y}} \right)} \right)$$

C1D represents one-dimensional convolution, *k* represents the size of the convolution kernel, *y* represents the input feature after global average pooling, and $\sigma$ passes through the sigmoid activation function to obtain the final weight *w*. The weights are multiplied by the corresponding elements of the original input feature to obtain the final output feature. After each layer of convolution, the elu [37] activation function is used to increase nonlinearity.

4 $${\text{elu}} = \left\{ {\begin{array}{*{20}l} {{\text{e}}^{{\text{x}}} - 1} \hfill & {x < 0} \hfill \\ x \hfill & {x \ge 0} \hfill \\ \end{array} } \right.$$

### Determining sub-regions containing insertion

Due to the limitations of alignment tools and sequencing technologies, there will be false alignments, which possibly cause some errors in the variant features. The length of each sub-region is only 200 bp, and the variation information may be expressed in adjacent regions. The continuous variant information of these sub-regions can be used to detect insertions. For example, the adjacent sub-regions of some insertion variations also contain soft-clipped breakpoint information. Therefore, it is crucial to capture the association among continuous sub-regions. INSnet uses a two-layer bidirectional GRU neural network [32], which solves the long-term dependence of traditional recurrent neural networks (RNNs) [38] and has fewer parameters and less computation than the commonly used LSTM [39].

Finally, the prediction is made through three fully connected layers, and the dropout layer is set. The last fully connected layer adopts a sigmoid activation function. If the predicted result is greater than 0.5, the sub-region is inferred to contain an insertion, and if it is less than 0.5, the sub-region is normal.

### Estimating the insertion site and length

After predicting whether a sub-region contains an insertion through previous steps, INSnet determines the precise site and length of the insertion. First, INSnet traverses all CIGAR strings in the alignments around this sub-region and saves the position where I ≥ 50 bp as a sub-insertion triple (Chr, Ref_start_, SV_len_). Chr indicates which chromosome it belongs to, and Ref_start_ indicates the insertion site in the reference. SV_len_ indicates the length of the insertion. Because there are usually many long reads aligned in the sub-region, we can obtain multiple sub-insertion triples.

In addition, due to sequencing errors and alignment tool bias, a large insertion may be split into multiple smaller parts aligned in the sub-region. Therefore, if the distance between two sub-insertion triples is smaller than 30 bp, they are merged into one large insertion region. For example, for two sub-insertion triples (Chr1, Ref_start1_, SV_len1_) and (Chr2, Ref_start2_, SV_len2_), if chr1 and chr2 belong to the same chromosome and Ref_start2_ − Ref_start1_ < 30, then a new sub-region triple is constructed: (Chr1, Ref_start1_, Ref_start2_ − Ref_start1_ + SV_len2_).

**Fig. 2:**
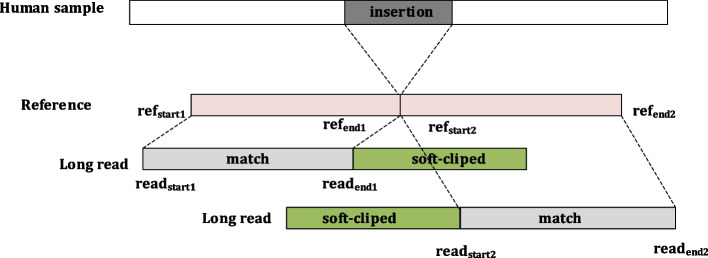
An example of soft-clipped alignment. There is an insertion in the human sample. One long read has two soft-clipped alignments. For this long read, the region [ref_start1_ ref_end1_] in the reference is aligned with the region [read_start1_, read_end1_] in it, and the region [ref_start2_ ref_end2_] in the reference is aligned with the region [read_start2_, read_end2_] in it. The two green parts represent soft-clipped

Next, for a soft-clipped long read in the sub-region, INSnet records the information as a hextuple sub-seg(Chr, Ref_start_, Ref_end_, Read_start_, Read_end_, stands). Chr indicates which chromosome it belongs to. Ref_start_ and Ref_end_ indicate the starting and ending positions in the reference. Read_start_ and Read_end_ indicate the starting and ending positions in the read. An example is shown in Fig. 2.

If one long read has two soft-clipped alignments, the two alignments have the same chromosome and the same direction and the following formula is satisfied, the read is considered to be a potential insertion region.

5 $$\left( {{\text{Ref}}_{{{\text{end}}1}} - {\text{Ref}}_{{{\text{start}}2}} } \right) - \left( {{\text{Read}}_{{{\text{end}}1}} - {\text{Read}}_{{{\text{start}}2}} } \right) \ge 50$$

At this time, both Ref_end1_ and Ref_start2_ may be the sites of insertion. INSnet then detects whether they are in the sub-region containing the insertion predicted by the neural network. Either Ref_end1_ or Ref_start2_ belongs to the predicted sub-region, and it is recorded as an insertion.

The potential insertions found above are all recorded in the form of triples (Chr, Ref_start_, SV_len_). If the distance between two triples is less than 1500 bp, the two triples are considered to be the same insertion and are stored in the same cluster. After processing all the triples, INSnet can obtain multiple clusters, and one cluster corresponds to an insertion. The insertion site is the median value of Ref_start_ in one cluster, and the length of the insertion is the median value of SV_len_ in this cluster.

### Model training

To train the model, we should know the exact site of insertion in the reference. The Genome in a Bottle Consortium (GIAB) [40] has supplied the sites of SVs for HG002, which have been widely used in other SV callers based on deep learning. The sites and types of SVs are available from https://ftptrace.ncbi.nlm.nih.gov/giab/ftp/data/AshkenazimTrio/analysis/NIST_SVs_Integration_v0.6/HG002_SVs_Tier1_v0.6.vcf.gz. We extract only insertion sites from this file for training INSnet.

First, we will align the CLR dataset and Nanopore dataset against the reference genome and generate the alignment matrix for each sub-region. If one alignment matrix corresponds to an insertion site, it is labelled 1; otherwise, it is labelled 0. Next, one hundred continuous alignment matrixes will be treated as one sample. After obtaining all samples, we divide the samples into a training set, test set and validation set. The samples of chromosomes 1–10 for HG002 are used as the training set, the samples of chromosome 11 are used as the validation set, and the remaining samples of chromosomes 12–22 are used as the test set. After completing training, INSnet can be validated on the test set. The SV sites and types of NA19240 are also available from NCBI dbVAR: https://ftp.ncbi.nlm.nih.gov/pub/dbVar/data/Homo_sapiens/by_study/vcf. The samples of chromosomes 1–22 for NA19240 are only used for testing. When using the CLR or Nanopore dataset to call insertions, users can use this trained model.

Because the characteristics of the CCS dataset differ from those of the CLR and Nanopore datasets, we generate another training set, validation set and test set by the method described above. When using the CCS dataset to call insertions, this trained model is adopted.

We use a computer with the following configuration for model training: 12-core 24-thread CPU (Intel(R) Xeon(R) Silver 4214 CPU @ 2.20 GHz) and an RTX 3090 graphics card.

### Model prediction

To ensure that as many insertions as possible are found, when using the deep learning network for insertion prediction, we make four sets predictions. INSnet will set a sliding window (default 50 bp) and perform prediction four separate times. For example, the first prediction is for sub-regions [0, 200], [200, 400], and [400,600]. The sub-regions in the second prediction are [50, 250], [250, 450] and [450, 650]. The sub-regions in the third prediction are [100,300], [300,500], and [500,700], and the sub-regions in the fourth prediction are [150,350], [350,550], and [550,750]. We keep the sub-regions that are considered to contain an insertion in any prediction.

## Results

INSnet benchmarks three state-of-the-art SV callers, Sniffles (2.0.6), SVIM (1.4.2), and CuteSV (1.0.13). Truvari (1.2.0) [41] is used to validate the results and obtain the evaluation metrics: recall, precision and F1 scores. Well-studied samples of HG002 and NA19240 are used for training and testing. For HG002, we use CLR and CCS data of Pacific Biosciences and sequencing data from Oxford Nanopore Technologies. For NA19240, we use CLR sequencing data. To test the sensitivity of SV callers for different sequencing coverage levels, we downsample the HG002 CLR data to obtain 35X, 20X, 10X, and 5X datasets. HG002 CCS data are downsampled to 10X and 5X datasets. We downsample HG002 ONT data to obtain 20X, 10X, and 5X datasets. For INSnet, SVIM and cuteSV, we set different support read parameters for different coverage. For the CLR and ONT datasets, support read is set to 10 for datasets with coverage greater than 40X. For the 35X, 20X, 10X, and 5X datasets, the support read is set to 5, 4, 3, and 2, respectively. For the 28X, 10X, and 5X CCS datasets, the support read is set to 3, 2, and 1, respectively. The details of the datasets are shown in the Additional file 1: Table S1.

### HG002 evaluation results for different callers based on the CLR dataset

First, we benchmark Sniffles, SVIM, cuteSV, and INSnet on the test data (chromosomes 12–22 for HG002) using the CLR dataset. The benchmark result is shown in Table 1.

**Table 1:** Performance comparison of SV callers on CLR dataset about HG002

Coverage	INSnet	cuteSV	SVIM	Sniffles
*CLR*				
69X				
Precision	0.9346	0.9247	0.9421	0.5849
Recalll	0.915	0.879	0.7967	0.8876
F1	0.9247	0.9012	0.8633	0.7051
35X				
Precision	0.9095	0.9194	0.9204	0.5806
Recall	0.8644	0.8596	0.7961	0.8741
F1	0.8864	0.8762	0.8538	0.6977
20X				
Precision	0.9084	0.8386	0.8366	0.5169
Recall	0.7838	0.8107	0.7547	0.8402
F1	0.8415	0.81	0.7936	0.64
10X				
Precision	0.8822	0.9502	0.934	0.2906
Recall	0.6122	0.575	0.5223	0.7149
F1	0.7228	0.7062	0.6715	0.4132
5X				
Precision	0.7643	0.6996	0.5988	0.4264
Recall	0.3943	0.4271	0.3911	0.4164
F1	0.5202	0.5201	0.4732	0.4213

INSnet has the highest recall and F1 score for all datasets with different coverage levels. For the 69X dataset, the F1 score is 2.3% higher than the second best score. This proves that INSnet can have a good effect when coverage is high. To examine the performance of callers on datasets with different coverage levels, we randomly downsample the HG002 CLR data to test sensitivity under different coverage levels. On the 35× dataset, INSnet’s F1 score is improved by 1% over that of cuteSV. At the same time, the recall is only less than 1% lower than that of Sniffles, but the precision of Sniffles is only 58.06%, while INSnet’s precision is as high as 90.95%. On the 20× dataset, INSnet's F1 score is 3% higher than that of cuteSV and has the highest precision. On the 10X dataset, INSnet's F1 score is 1.66% higher than that of cuteSV. Although Sniffles has the highest recall, the precision is only 29%. INSnet has the second highest recall and high precision. Due to the high error rate of long read, more reads are needed to ensure the accuracy of data and call SV. Therefore, as coverage decreases, it is difficult to distinguish false SVs generated by sequencing errors from true SV. At the same time, the variation information contained in the low coverage data is not obvious, and the detection of insertion becomes more difficult. Common SV caller, such as Sniffles, SVIM, CuteSV will also encounter the same problem. Therefore, in the 10X data, the results obtained by INSnet is low. On the 5× dataset, although the F1 score of INSnet is similar to that of cuteSV, the precision is much higher than that of cuteSV. This proves that INSnet can achieve good results with CLR data under different coverage levels.

### HG002 evaluation results for different callers based on the ONT dataset

We further benchmark Sniffles, SVIM, cuteSV, and INSnet on the test data (chromosomes 12–22) using the ONT dataset. The benchmark result is shown in Table 2. INSnet achieves the highest F1 score at 48 × coverage and has the highest precision. Then, we also randomly downsample the ONT data to 20X, 10X, and 5X coverage. On the 20× data, INSnet has the highest F1 score and the highest precision. On the 10× and 5× data, the INSnet results are only slightly worse than those from Sniffles. This proves that INSnet can also perform well on ONT data. Due to the different sequencing technologies of Oxford Nanopore Technologies (ONT) and PacBio Continuous Long Reads (CLR), the accuracy of the data is also different. Compared with CLR data, ONT data has a higher sequencing error rate [42], which possibly infect the accuracy of the feature extracted from alignment file by INSnet In the high-coverage ONT data, INSnet achieved the highest F1 score in the coverage of 48× and 20×. Insertion detection becomes more difficult as data coverage decreasing.

**Table 2:** Performance comparison of SV callers on ONT dataset about HG002

Coverage	INSnet	cuteSV	SVIM	Sniffles
*ONT*				
48X				
Precision	0.9012	0.8865	0.7883	0.8889
Recall	0.8585	0.8531	0.8273	0.8693
F1	0.8793	0.8695	0.8073	0.879
20X				
Precision	0.8893	0.8615	0.6754	0.8419
Recall	0.8467	0.83	0.8316	0.8623
F1	0.8675	0.8455	0.7454	0.852
10X				
Precision	0.8827	0.87	0.7306	0.8243
Recall	0.7531	0.7488	0.7133	0.8305
F1	0.8128	0.8049	0.7218	0.8274
5X				
Precision	0.8519	0.8423	0.6937	0.8645
Recall	0.6628	0.6321	0.6213	0.6659
F1	0.7232	0.7222	0.6555	0.7524

### NA19240 evaluation results about different callers based on CLR dataset

To test the performance of INSnet on other datasets, we also use the more challenging NA19240 dataset. The ground-truth call sets are collected from the NCBI dbVAR database. The results are shown in Table 3. On the NA19240 dataset, INSnet has the highest recall and F1 scores, where the recall is 2.79% higher than the second best (cuteSV) and the overall F1 is 1% higher than that of cuteSV. This shows that INSnet can have good sensitivity for different datasets and can achieve better results than other tools.

**Table 3:** The performance on CLR dataset about NA19240

Coverage	INSnet	cuteSV	SVIM	Sniffles
*CLR*				
41X				
Precision	0.4719	0.6276	0.4163	0.4679
Recalll	0.2033	0.1754	0.0305	0.1754
F1	0.2842	0.2742	0.0568	0.2551

### The performance for insertions with different length

To validate the performance of SV callers for insertions with different lengths, we classify the insertions into five intervals, [50, 200], [200, 500], [500, 1000], [1000, 5000], and [5000,]. The insertion benchmark results with different lengths for the 69X dataset are shown in Table 4. INSnet can achieve above-average F1 scores in each interval. Among them, in the insertion interval of 1000–5000, the F1 score of INSnet (0.9142) is 14% higher than that of the second-best tool, cuteSV (0.7735). The recall of INSnet (0.9142) is 26% higher than that of the second-best, cuteSV (0.6524). In the interval greater than 5000, Sniffles, SVIM, and cuteSV all find few variant sites. The recall of Sniffles is 0.2292, but its precision is only 0.0399. The precision of cuteSV reaches 1, but the recall is only 0.1458. The precision of INSnet reaches 0.8888, the recall reaches 0.5, and the F1 score is nearly 40% higher than the second best score. Experiments show that INSnet can obtain good results in each interval, especially in detecting large insertions.

**Table 4:** The performance of insertions in different sizes on 69× data about HG002

Phase	INSnet	cuteSV	SVIM	Sniffles
*CLR69X*				
50–200				
Precision	0.8961	0.8639	0.8958	0.3106
Recall	0.8817	0.9458	0.9421	0.9126
F1	0.8888	0.903	0.9184	0.463
200–500				
Precision	0.9337	0.9506	0.6964	0.9108
Recall	0.9173	0.9155	0.8803	0.9525
F1	0.9254	0.9327	0.7776	0.9312
500–1000				
Precision	0.8276	0.9207	0.9226	0.8617
Recall	0.8484	0.7626	0.7222	0.8182
F1	0.8379	0.8343	0.8102	0.8394
1000–5000				
Precision	0.9142	0.95	0.9592	0.2327
Recall	0.9142	0.6524	0.2017	0.4893
F1	0.9142	0.7735	0.3333	0.3154
5000-				
Precision	0.8888	1	0	0.0399
Recall	0.5	0.1458	0	0.2292
F1	0.64	0.2545	0	0.0679

### The influence of support read on INSnet

We further evaluate the parameter, support read, for INSnet on the CLR 69X dataset about HG002. The support read parameter refers to the minimum support reads of the insertion to be called. As shown in Table 5, setting different values of support read will result in different precision, recall, and F1 score. When the support read is larger, the sensitivity is reduced and the accuracy is improved. When the support read is smaller, accuracy decreases and sensitivity increases.

**Table 5:** The performance of different support read on hg002 69X data

Coverage	Support ≥ 1	Support ≥ 3	Support ≥ 5	Support ≥ 7	Support ≥ 10
*CLR*					
69X					
Precision	0.5934	0.7235	0.8267	0.8877	0.9346
Recalll	0.9279	0.9274	0.9263	0.9225	0.915
F1	0.7239	0.8128	0.8737	0.9048	0.9247

### HG002 evaluation results about different callers for CCS dataset

Due to its high accuracy for the CCS data, INSnet is specifically trained based on the CCS dataset. We also benchmark Sniffles, SVIM, cuteSV, and INSnet on the test data (chromosomes 12–22) by using CCS datasets. We randomly downsample the CCS dataset to 10X and 5X coverage. Table 6 shows that INSnet performs the best on all three datasets. For the 28× dataset, the F1 score improves by 0.7% over that of Sniffles, and INSnet has the highest precision. For the 10× data, the F1 score improves by nearly 1.5% over that of Sniffles. On the 5× data, the F1 score is 4% better than that of SVIM. This proves that INSnet has good performance for CCS datasets with different coverage.

**Table 6:** Performance comparison of SV callers on CCS data about HG002

Coverage	INSnet	cuteSV	SVIM	Sniffles
*CCS*				
28X				
Precision	0.9215	0.893	0.8735	0.9001
Recall	0.922	0.9247	0.9139	0.9301
F1	0.9218	0.9086	0.8933	0.9148
10X				
Precision	0.905	0.8881	0.8629	0.8945
Recall	0.887	0.8499	0.8666	0.8714
F1	0.896	0.8686	0.8647	0.8828
5X				
Precision	0.8969	0.8281	0.8001	0.911
Recall	0.8327	0.8825	0.8419	0.6993
F1	0.8636	0.78	0.8204	0.7912

### The model classification result

We evaluated the classification ability of the model. For HG002, we alse use CLR, ONT and CCS data. And we randomly downsample the HG002 CLR data to obtain 35X, 20X, 10X, and 5X datasets. HG002 CCS data are downsampled to 10X and 5X datasets. We downsample HG002 ONT data to obtain 20X, 10X, and 5X datasets. The detailed experimental results are provided in the Additional file 1: Tables S2, S3 and S4.

## Discussions and conclusion

In this study, we developed INSnet, a deep learning-based method for detecting insertions. INSnet collects different features in the alignments between long reads and a reference genome, analyses the features through depthwise separable convolution and two attention mechanisms, and then uses a bidirectional GRU network and fully connected layers to determine the sub-regions that contain an insertion. We test the performance of INSnet on several datasets and compare it with three state-of-the-art tools. INSnet can find insertions with good performance on different datasets and under different coverage levels.

In this paper, we consider only insertions, but there are other types of variations, such as deletions, inversions, and copy number variations, that cannot be called. In addition, INSnet is currently unable to call genotypes. We will address these issues in future work.

## Supplementary Information

**Additional file 1.** Supplementary Tables and Figures.

## Data Availability

The HG002 data can be downloaded from https://ftp.ncbi.nih.gov/giab/ftp/data/AshkenazimTrio. The high confidence callset and the high confidence regions of HG002 were provided by GIAB and downloaded from https://ftp-trace.ncbi.nlm.nih.gov/giab/ftp/data/AshkenazimTrio/analysis/NIST_SVs_Integration_v0.6/HG002_SVs_Tier1_v0.6.vcf.gz. The alignment files of samples NA19240 can be downloaded from http://ftp.1000genomes.ebi.ac.uk/vol1/ftp/data_collections/hgsv_sv_discovery/working/20160905_smithm_pacbio_aligns/NA19240_bwamem_GRCh38DH_YRI_20160905_pacbio.bam. The source code is available from GitHub at https://github.com/eioyuou/INSnet.

## Abbreviations

BAM: Binary sequence alignment
GIAB: Genome in a Bottle Consortium
DSC: Depthwise separable convolution
Bi-GRU: Bidirectional gated recurrent unit
CBAM: Convolutional block attention module
ECA: Efficient channel attention
SNPs: Single nucleotide polymorphisms
INDELs: Small insertion and/or deletion
SVs: Structural variations
