# Supplementary material for: INSnet: a method for detecting insertions based on deep learning network
Source: BMC Bioinformatics. 2023 Mar 6;24:80. doi: 10.1186/s12859-023-05216-0 (PMC9990265; doi:10.1186/s12859-023-05216-0)
Supplement: Supplementary file 1 — Additional file 1. Supplementary Tables and Figures. [file 12859_2023_5216_MOESM1_ESM.docx]

**Supplementary Materials**

INSnet: a method for detecting insertions based on deep learning network

**1 The details of the datasets**

The details of all datasets used in this paper is shown in Table S1.

**Table S1. The details of the datasets.**

|  | HG002 CLR | HG002 CCS | HG002 ONT | NA19240 CLR |
| --- | --- | --- | --- | --- |
| Read Count | 2915733 | 6596012 | 19328993 | 20452822 |
| Average Length | 7938 | 13478 | 8098 | 6503 |
| Coverage | 69X | 28X | 48X | 41X |

**2 The module classification result of HG002**

IWhen using deep learning module to classify the sub-regions, the classification accuracy is also important for the following steps. The details of classification results are shown in the following Tables S2-S4. In addition to accuracy, we also added true positives (TP), false positives (FP), true negatives (TN), false negatives (FN) as metrics. False negatives and false positives are samples that were incorrectly classified. True negatives and true positives are samples that were correctly classified. AUC[31] refers to the performance of a classification model at all classification thresholds

**Table S2. The classification result on CLR datasets.**

| data | | accuracy | AUC | TP | FP | TN | FN |
| --- | --- | --- | --- | --- | --- | --- | --- |
| CLR | 69x | 0.9981 | 0.8849 | 5163 | 1165 | 4049087 | 6585 |
|  | 35x | 0.998 | 0.8731 | 4739 | 1071 | 4049180 | 7010 |
|  | 20x | 0.9979 | 0.8579 | 4195 | 892 | 4048361 | 7552 |
|  | 10x | 0.9977 | 0.828 | 3311 | 774 | 4044491 | 8424 |
|  | 5x | 0.9976 | 0.7977 | 2337 | 538 | 4004917 | 9208 |

**Table S3. The classification result on ONT datasets.**

| data | | accuracy | AUC | TP | FP | TN | FN |
| --- | --- | --- | --- | --- | --- | --- | --- |
| ONT | 50 | 0.9981 | 0.86 | 5462 | 1281 | 4048970 | 6287 |
|  | 20x | 0.998 | 0.8564 | 5111 | 1433 | 4048818 | 6638 |
|  | 10x | 0.9979 | 0.8434 | 4356 | 1322 | 4048928 | 7394 |
|  | 5x | 0.9976 | 0.8133 | 3280 | 1060 | 4019264 | 8396 |

**Table S4. The classification result on CCS datasets.**

| data | | accuracy | AUC | TP | FP | TN | FN |
| --- | --- | --- | --- | --- | --- | --- | --- |
| CCS | 28x | 0.9981 | 0.8308 | 6381 | 2299 | 4044964 | 5356 |
|  | 10x | 0.998 | 0.83 | 6219 | 2620 | 4042655 | 5506 |
|  | 5x | 0.9979 | 0.8221 | 5822 | 2649 | 4003842 | 5687 |

**3 The example of soft-clipped alignment**

**Figure S1. An example of left soft-clipped alignment.** There is an insertion in the human sample, region A and region C are aligned with reference. For this long read sequenced from human sample, the region C in the reference is aligned with the region ML, and the region SL cannot be aligned with the reference. The breakpoint divided this long read into two different parts.

**Figure S2. An example of right soft-clipped alignment.** There is an insertion in the human sample, region A and region C are aligned with reference. For this long read, the region C in the reference is aligned with the region MR, and the region SR cannot align with the reference. The right soft-clip breakpoint divided this long read into two different parts.
